# Supplementary material for: Orientation dependent molecular electrostatics drives efficient charge generation in homojunction organic solar cells
Source: Nat Commun. 2020 Sep 15;11:4617. doi: 10.1038/s41467-020-18439-z (PMC7494863; doi:10.1038/s41467-020-18439-z)
Supplement: Supplementary file 1 — Supplementary information [file 41467_2020_18439_MOESM1_ESM.pdf]

Supplementary Information for

Orientation Dependent Molecular Electrostatics Drives Efficient Charge Generation in Homojunction Organic Solar Cells

Yifan Dong[1,a], Vasileios C. Nikolis[2,a,b], Felix Talnack[3], Yi-Chun Chin[4], Johannes Benduhn[2], Giacomo Londi[5], Jonas Kublitski[2], Xijia Zheng[1], Stefan C. B. Mannsfeld[3], Donato Spoltore[2], Luca Muccioli[6], Jing Li[7], Xavier Blase[7], David Beljonne[5], Ji-Seon Kim[4,*], Artem A. Bakulin[1], Gabriele D'Avino[7,*], James R. Durrant[1,8,*], Koen Vandewal[9,*]

[1]Department of Chemistry, Centre for Processable Electronics, Imperial College London, London, United Kingdom W12 0BZ

[2]Dresden Integrated Centre for Applied Physics and Photonic Materials (IAPP) and Institute for Applied Physics, Technische Universität Dresden, Nöthnitzer Str. 61, 01187 Dresden, Germany

[3]Center for Advancing Electronics Dresden (cfaed) and Faculty of Electrical and Computer Engineering, Technische Universität Dresden, Helmholtzstr. 18, 01069 Dresden, Germany

[4]Department of Physics, Imperial College London, London, United Kingdom SW7 2AZ

[5]Laboratory for Chemistry of Novel Materials, University of Mons, Place du Parc 20, 7000 Mons, Belgium

[6]Department of Industrial Chemistry, University of Bologna, Viale Risorgimento 4, 40136 Bologna, Italy

[7]Institut Neél, CNRS and Grenoble Alpes University, Rue des Martyrs 25, 38042 Grenoble, France

[8]SPECIFIC, College of Engineering, Swansea University, Bay Campus, Swansea SA1 8EN, United Kingdom

[9]Institute for Materials Research (IMO-IMOMEC), Hasselt University, Wetenschapspark 1, 3590 Diepenbeek, Belgium

[a]These authors contributed equally: Yifan Dong, Vasileios C. Nikolis

[b]Current address: Heliatek GmbH, Treidlerstraße 3, 01139 Dresden, Germany

*email: koen.vandewal@uhasselt.be; j.durrant@imperial.ac.uk; gabriele.davino@neel.cnrs.fr ; ji-seon.kim@imperial.ac.uk

Supplementary Figures

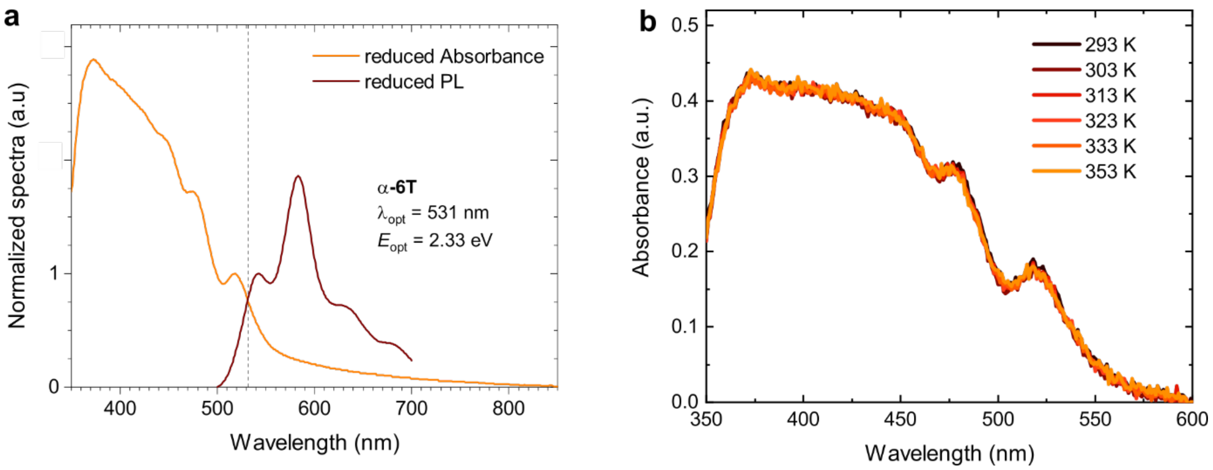

Supplementary Figure 1. a, Reduced absorbance and photoluminescence spectra of the α-6T thin film with mixed orientation. The intersection provides the optical gap (E_{opt})[14] which is equal to 2.33 eV. **b,** Absorbance spectra of the same α-6T thin film measured at different temperatures. No thermal artefacts were observed in this film when carrying out the absorption measurement at higher temperatures.

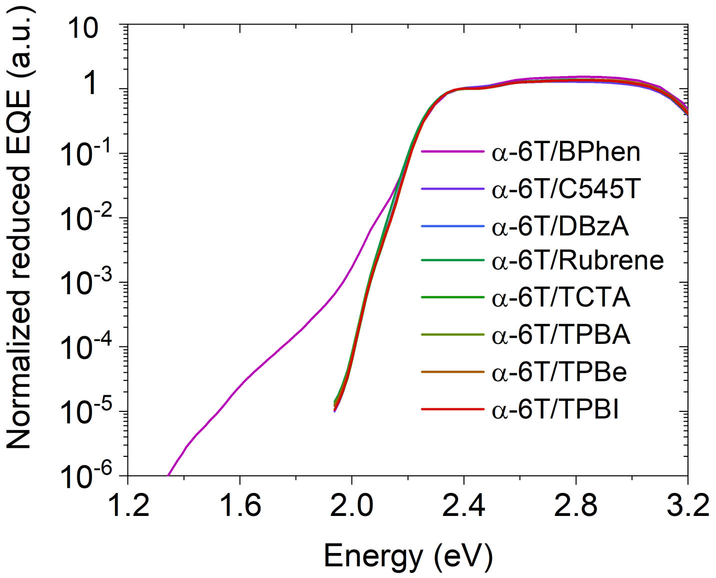

Supplementary Figure 2. Sensitively measured external quantum efficiency (sEQE) spectra of devices employing α-6T with various buffer layers. No subgap absorption features are observed, apart from the case with BPhen.

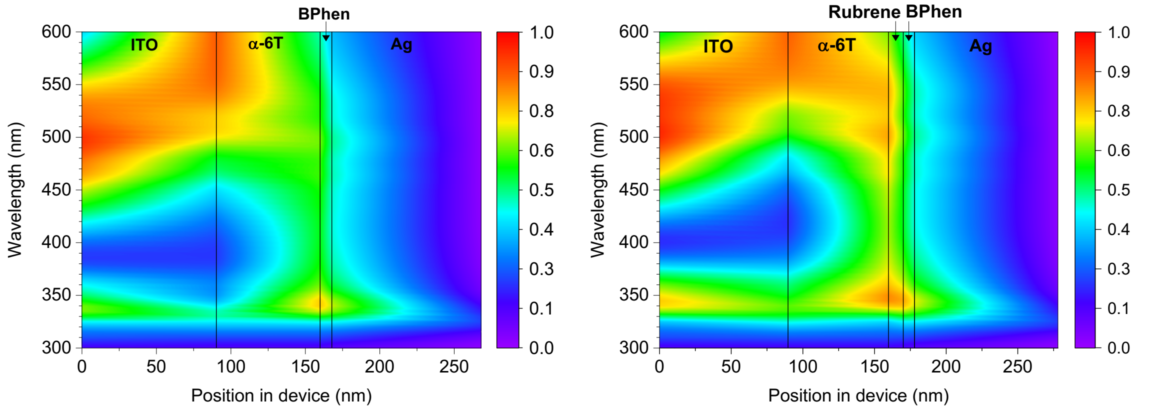

Supplementary Figure 3. Distribution of optical field in two α-6T based homojunction solar cells employing either BPhen, or Rubrene and BPhen between α-6T and the Ag contact. Simulations were performed based on transfer matrix method and show that for both devices the absorption of photons, relevant for the external quantum efficiency spectra shown in Figure 1 of the manuscript, occurs mostly in the bulk of α-6T close to the interface with ITO.

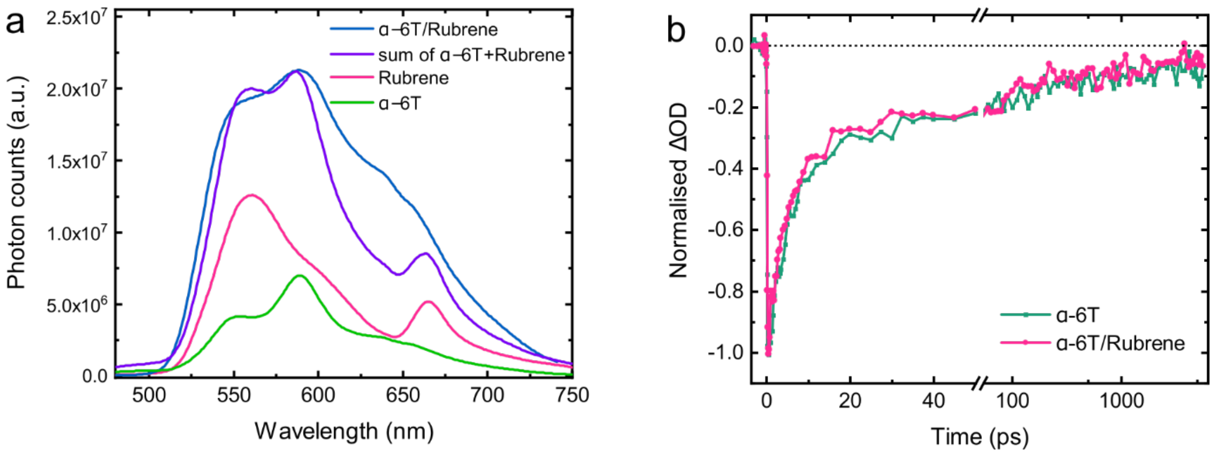

Supplementary Figure 4. Spectroscopic characterisation comparing α-6T, rubrene, and α-6T/rubrene films. **a**, Photoluminescence (PL) spectra for neat α-6T, neat rubrene and α-6T/rubrene thin films following excitation at 450 nm. The PL of α-6T/rubrene resembles the sum of that in the neat α-6T and neat rubrene. **b,** Transient absorption kinetics at 593 nm for the neat α-6T and α-6T/rubrene thin films. These two kinetics decay with the same lifetime, implying that the α-6T/rubrene interface has no effect in dissociating excitons and hence does not lead to charge generation.

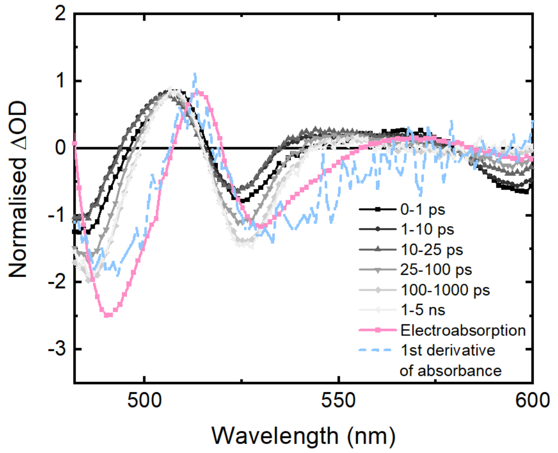

Supplementary Figure 5. Comparing normalised transient absorption (TA) spectra with the electroabsoption spectra measured independently for α-6T homojunction solar cell device and the first derivative of the absorbance of α-6T.

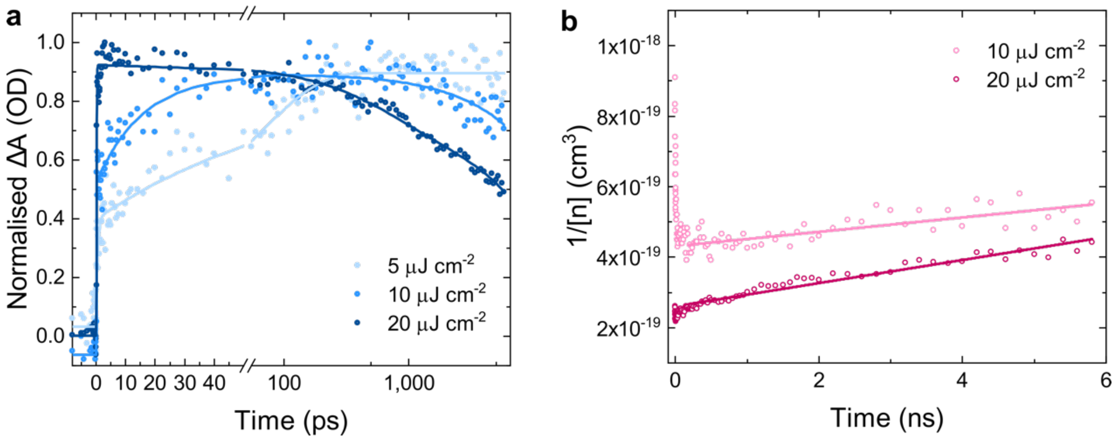

Supplementary Figure 6. Transient absorption (TA) kinetics for the charges (probed at 780 nm) in α-6T thin film with mixed orientations. **a,** The sample was excited at various fluences including 5, 10 and 20 μJ cm^{-2} with a pump wavelength of 450 nm. The kinetics exhibit strong dependence on the fluence. The higher the number of carriers excited, the faster the decay. At 5 μJ cm^{-2}, there is negligible bimolecular recombination. **b,** Fitting Analysis of bimolecular recombination rate constants from the TA decay representing the bimolecular recombination of charges. For a bimolecular recombination following the rate equation of $\frac{d[n]}{dt} = -kn^2$, the rate constant (k) can be extracted from the slope by plotting $\frac{1}{[n]}$ against t as indicated in the figure. The solid lines are the linear fitting to the raw data. In this case, the slope was fitted to be 1.9×10^{-11} cm^3 s^{-1} and 3.3×10^{-11} cm^3 s^{-1} respectively under pump fluences of 10 and 20 μJ cm^{-2}.

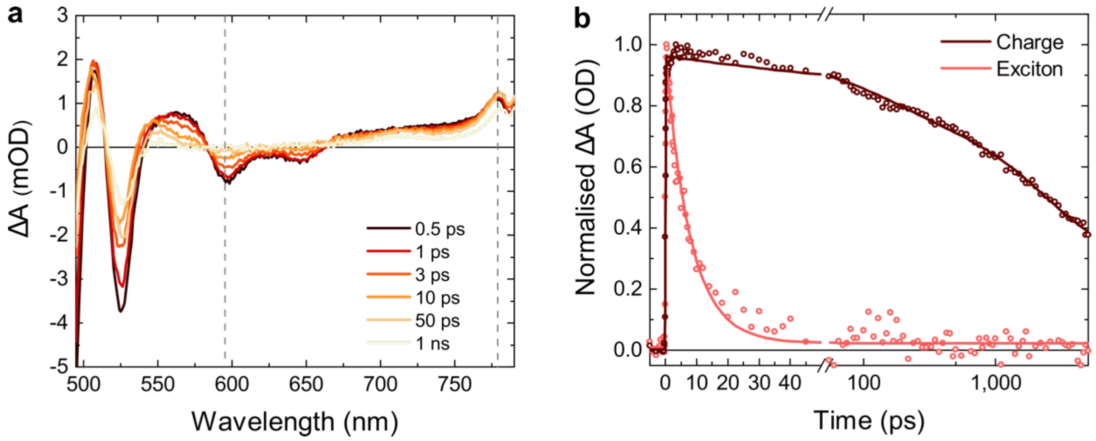

Supplementary Figure 7. Transient absorption characterisation for the α-6 -based homojunction organic solar cells (HOSCs) with mixed orientations. **a,** Transient absorption (TA) spectra for α-6T-based HOSC as a function of pump probe time delay. **b,** TA kinetics extracted from 593 nm and 780 nm where the former represents excitons dynamics and the latter represents the charge dynamics. The sample was measured under a pump excitation of 450 nm with a fluence of 20 μJ cm^{-2} in a reflectance geometry.

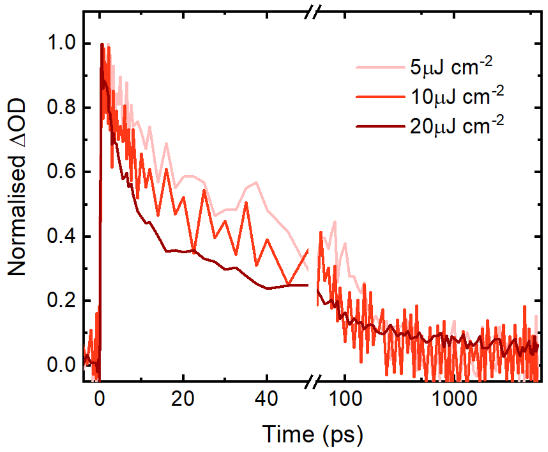

Supplementary Figure 8. Transient absorption kinetics for the excitons (probed at 593 nm) in α-6T thin film with mixed orientations. The sample was excited at various fluences including 5, 10 and 20 μJ cm^{-2} with a pump wavelength of 450 nm. The kinetics exhibit minor dependence on the fluence when it is below 10 μJ cm^{-2}. A low excitation fluence (5 μJ cm^{-2}) was employed to avoid the exciton-exciton annihilation and non-linear effects.

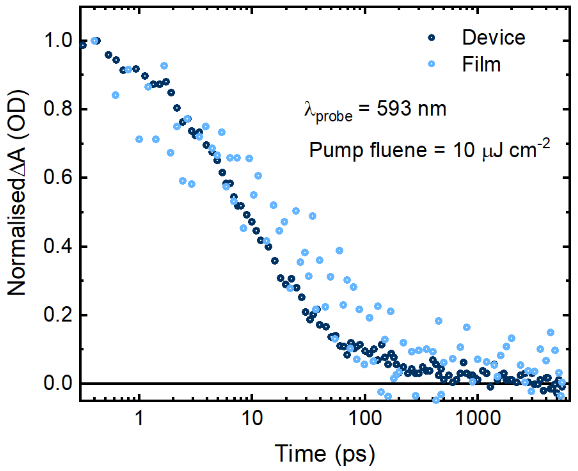

Supplementary Figure 9. Normalised transient absorption kinetics comparison for the excitons in α-6T thin film with device. The kinetics for excitons probed at 593 nm show a similar half-lifetime in both samples. Both samples were excited at 10 μJ cm^{-2}.

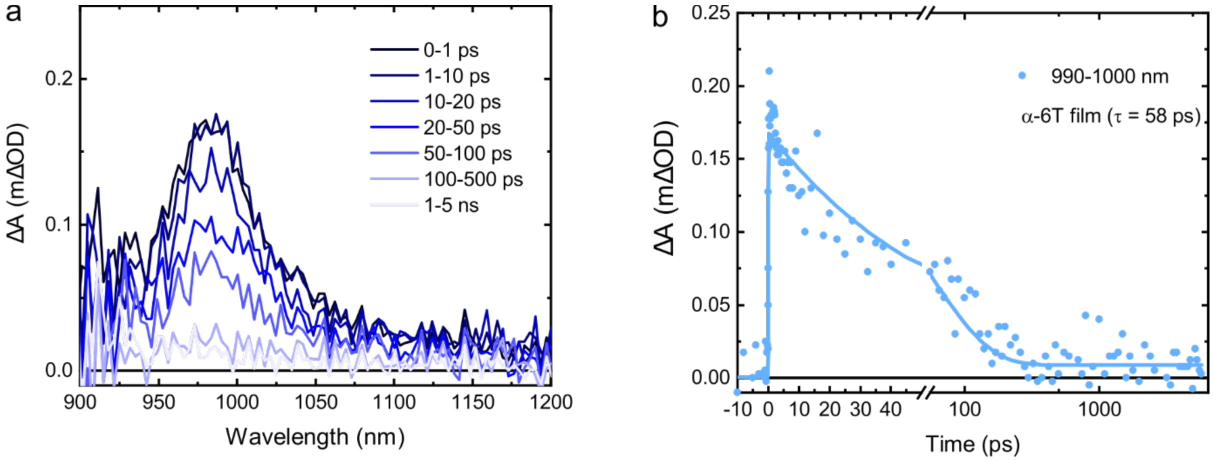

Supplementary Figure 10. Transient absorption spectroscopy characterisation for the α-6T thin film in the near-Infrared region. **a,** TA spectra as a function of pump-probe time delay. **b,** TA kinetics probed at 990-1100 nm. The solid line represents the mono-exponential fitting which gives a lifetime of 58 ps. The sample was excited at 450 nm with an excitation fluence of 10 μJ cm^{-2}.

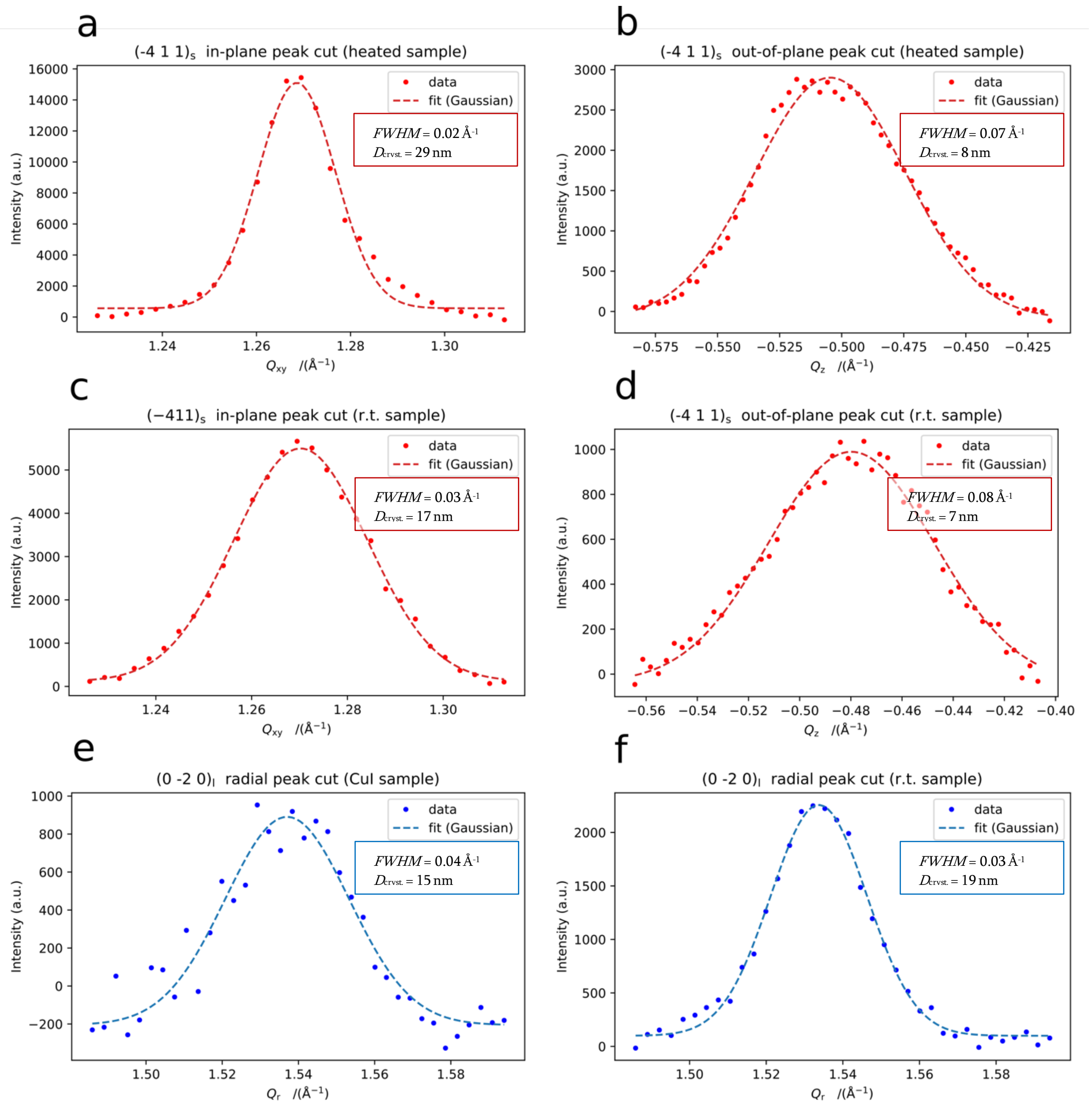

Supplementary Figure 11. Peak shapes extracted from the GIWAXS 2D images of the samples prepared at elevated temperatures, room temperature and on CuI, showing standing, mixed and lying molecular orientations, respectively. The profiles were extracted from peaks below the horizon, to minimize peak broadening effects stemming from sample size, as only scattering from the very end of the thin film contributes to those peaks. **a, b,** In- and out-of-plane profile of the (-4 1 1) peak for the sample prepared at elevated temperature exhibiting standing molecular orientations. **c, d,** In- and out-of-plane profile of the (-4 1 1) peak for the sample prepared at room temperature. This peak originates from the standing crystallites in the film with mixed orientations. **e, f,** Radial cut of the (0 -2 0) peak originating from the lying molecules in the CuI and the room temperature sample. Radial cuts were performed for e and d, because of the arching of the peaks originating from the lying molecules. The crystallite size

was calculated from the FWHM value by the Scherrer formula, $D_{hkl} = 2\pi K/\Delta q_{hkl}$, where Δq_{hkl} is the FWHM value of the specific peak and $K \approx 0.9$ the Scherrer constant.[15]

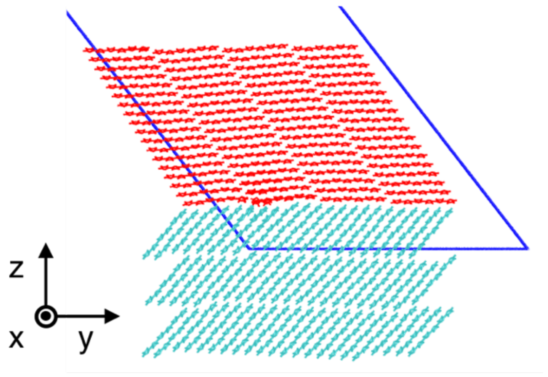

Supplementary Figure 12. Rendering of the α-6T standing/lying interface employed in electronic structure calculations. The sample counts 264 standing and 288 lying molecules. The blue frame shows the simulation box with periodic boundary conditions applied along the x and y directions.

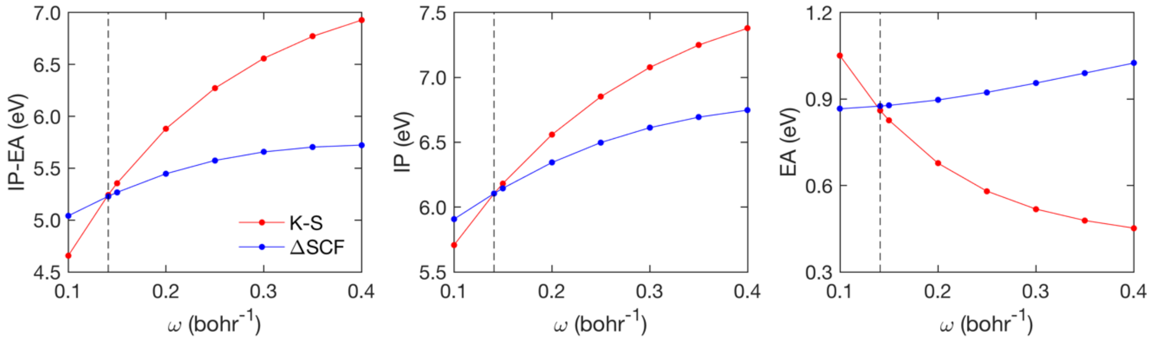

Supplementary Figure 13. Gap-tuning of the range-separation parameter ω of the density functional ωB97X. The optimal ω=0.141 bohr^{-1} (vertical line) corresponds to the value for which the Kohn-Sham HOMO-LUMO gap equals the difference between the ionization potential (IP) and the electron affinity (EA) obtained from total energy differences between neutral molecules and ions (ΔSCF). We note that the same value of ω permits also the matching of Kohn-Sham levels with IP and EA. These gas-phase DFT calculations employed the cc-pVTZ basis set.

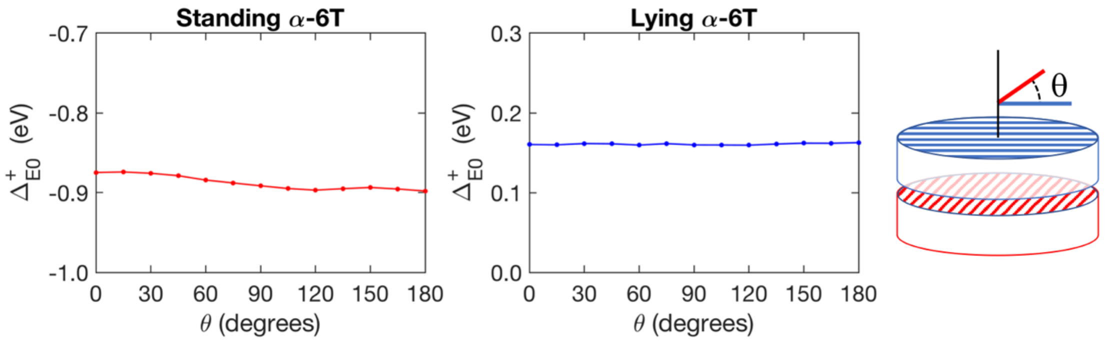

Supplementary Figure 14. Electrostatic energies of a hole in the standing and lying domain as a function of the relative in-plane orientations of the two subsystems, showing that the interface energetics is approximately independent on this structural parameter. These results were obtained with a non-polarizable point charge model. The right sketch illustrates the simulation sample setup: Two disk-shaped crystalline supercells (30 nm radius) of the laying and standing molecules were built as described in Supplementary Note 1 and placed at 4 Å distance from each other. One subsystem was then axially rotated by an angle θ. These structures were employed in electrostatic calculations without further structural relaxation.

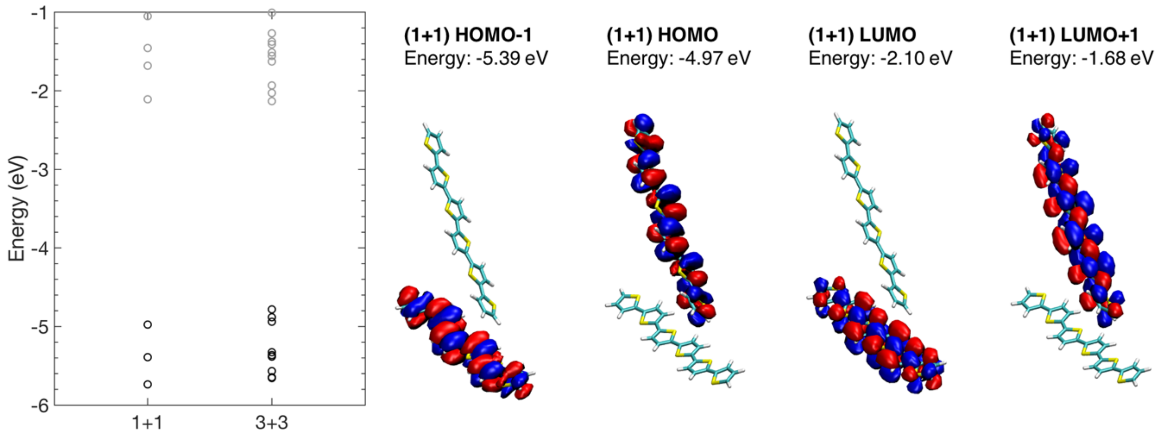

Supplementary Figure 15. Left: Quasiparticle energy levels from embedded (QM/MM) *GW* calculations for different size of the QM subsystem, i.e. “1+1” and “3+3”. Occupied and unoccupied levels are plot as black and gray circles, respectively. In the “3+3” system one can notice the formation of states manifolds due to charge delocalization effects. Right: Rendering of the frontier molecular orbitals of the “1+1” system that can be identified as the HOMO and LUMO levels of standing and lying molecules. The levels of standing and lying molecules are offset by 0.4 eV as a result of intermolecular electrostatic interactions. The orbitals of the “3+3” system (not shown) are consistent with those of the smaller one, with a highest-occupied (lowest-unoccupied) manifold of states delocalized over standing (lying) molecules.

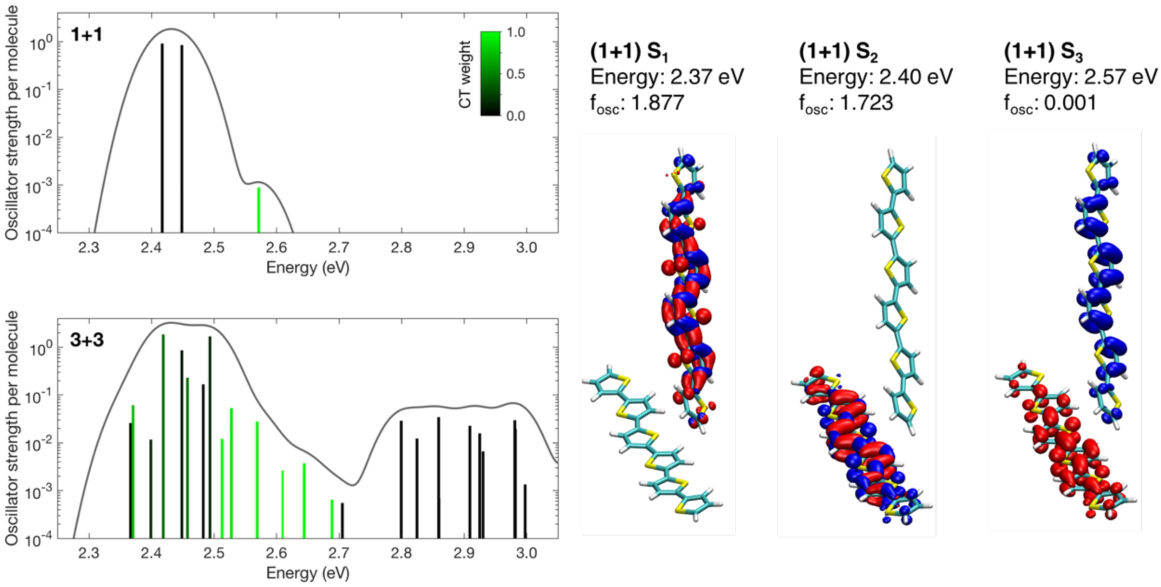

Supplementary Figure 16. Left: Comparison of the BSE/GW/MM absorption spectrum calculated for the "1+1" and "3+3" system, illustrating the effects of exciton and charge delocalization over several molecules. The bars colour quantifies the weight of inter-domain charge-transfer (CT) states in each excitation. Right: Electron-hole density plots of the 3 lowest-energy singlet excitations (S_n) for the "1+1" system. S_1 and S_2 corresponds to Frenkel excitons localized on the standing and lying molecule, respectively. S_3 is an inter-domain CT state.

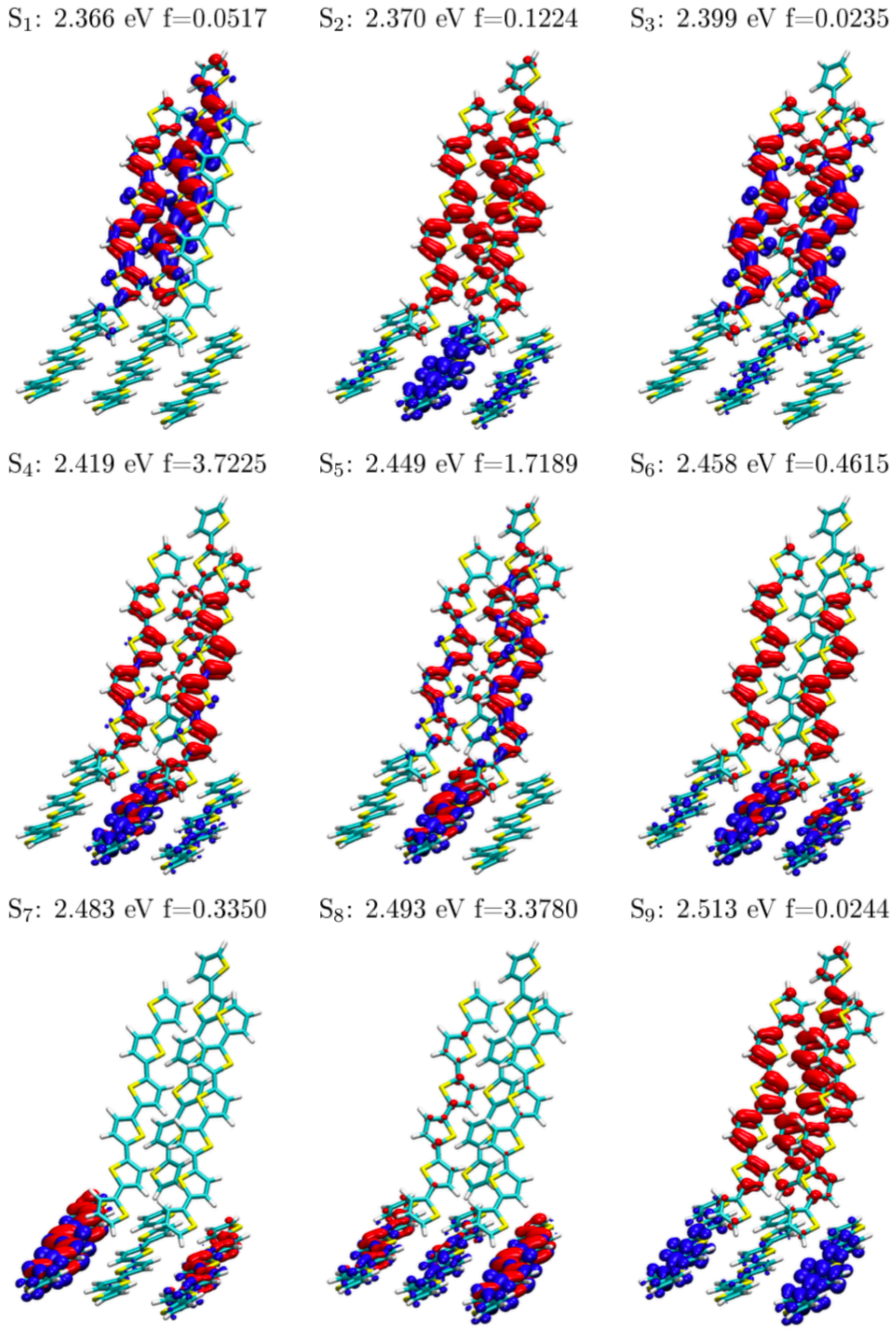

Supplementary Figure 17. Electron-hole density plots of singlet excitations calculated for the “3+3” system. States below 2.7 eV corresponds to Frenkel and inter-domain charge-transfer (CT) excitations with variable mixing proportions (see e.g. S_8, S_{10}, S_{12}). Excitations above 2.7 eV comprise intra-domain CT states (see e.g. S_{15}, S_{19}) and higher-energy Frenkel excitons (see e.g. S_{20}). Continues in Supplementary Figures 18, 19.

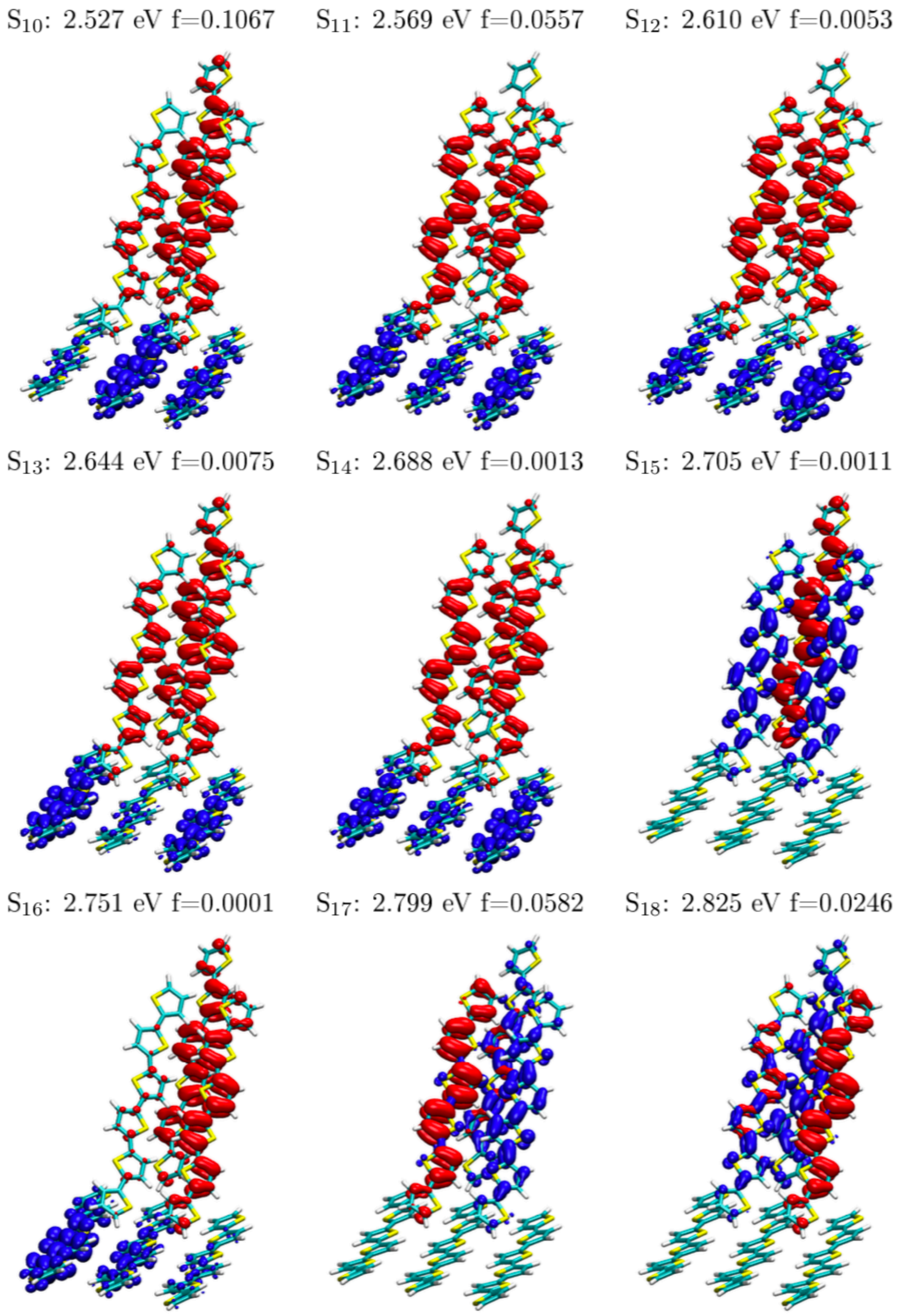

Supplementary Figure 18. Electron-hole density plots of singlet excitations calculated for the “3+3” system. Follow-up from Supplementary Figure 17.

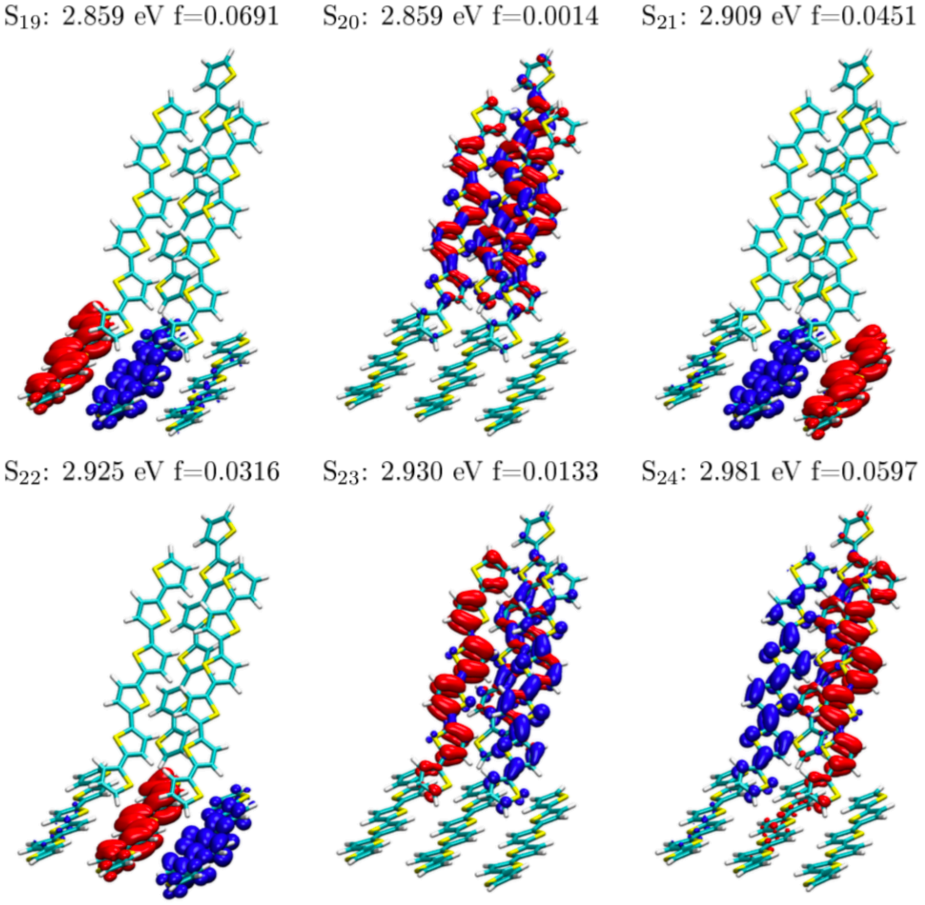

Supplementary Figure 19. Electron-hole density plots of singlet excitations calculated for the “3+3” system. Follow-up from Supplementary Figures 17, 18.

Supplementary Tables

Supplementary Table 1. Summary of the energy level values (for the lowest unoccupied and highest occupied molecular orbitals, LUMO and HOMO, respectively) of α-6T and the other buffer layer (BL) materials used in the investigated organic solar cells of this work. Values are based on literature and references are included in each case. Notwithstanding the dispersion of the values, the LUMO values of BL materials are in the range of those of α-6T.

Material	LUMO (eV)	HOMO (eV)	References
α-6T (α-sexithiophene)	2.6 – 3.1	5.1 – 5.4	[1–3]
BPhen (Bathophenanthroline)	2.9 – 3.0	6.2 – 6.7	[4–6]
Rubrene (5,6,11,12-Tetraphenyltetracene)	3.2	5.4	[7-8]
C545T (10-(2-Benzothiazolyl)-2,3,6,7-tetrahydro-1,1,7,7-tetramethyl-1H,5H,11H (1)benzopyropyrano(6,7-8-I,j) quinolizin-11-one)	2.8 – 3.1	5.5 – 5.6	[7–10]
DBzA (9,10-Bis[4-(6-Methylbenzothiazol-2-yl) phenyl] anthracene)	2.8	5.7	[8]
TCTA (Tris(4-carbazoyl-9-ylphenyl) amine)	2.3	5.7	[11]
TBPe (2,5,8,11-Tetra-tert-butylperylene)	2.7	5.4	[12]
TPBA (9,9‘,10,10‘ tetraphenyl-2 ,2‘ bianthracene)	2.8	5.8	[8]
TPBI (2,2',2-(1,3,5-Benzinetriyl)-tris(1-phenyl-1-H-benzimidazole))	2.7 – 2.8	6.2 – 6.7	[5,6,13]

Supplementary Table 2. Energy and voltage losses in the devices employing α-6T and various buffer layers. The voltage at the radiative limit (V_{rad}) is calculated based on the EQE spectra of the devices shown in Supplementary Figure 2, using the method outlined in ref. 16. The non-radiative voltage losses ($\Delta V_{non\text{-}rad}$) are estimated by subtracting the open-circuit voltage of the devices (V_{OC}) from V_{rad}. The total energy losses are estimated by subtracting the V_{OC} from the optical gap (E_g) of α-6T (2.33 eV). The lowest energy losses (0.72 eV) are observed for the device with rubrene, which provides the highest V_{OC} (1.61 V).

**Device structure**	V_{OC} **(V)**	V_{rad} **(V)**	$\Delta V_{non\text{-}rad}$	$E_g - qV_{OC}$ **(eV)**
ITO / α-6T / BPhen / Ag	1.25	1.58	0.33	1.08
ITO / α-6T / Rubrene / BPhen / Ag	1.61	1.87	0.26	0.72
ITO / α-6T / C545T / BPhen / Ag	1.46	1.89	0.40	0.87
ITO / α-6T / DBzA / BPhen / Ag	1.57	1.88	0.31	0.76
ITO / α-6T / TCTA / BPhen / Ag	1.57	1.88	0.31	0.76
ITO / α-6T / TPBA / BPhen / Ag	1.61	1.88	0.27	0.72
ITO / α-6T / TBPe / BPhen / Ag	1.41	1.88	0.47	0.92
ITO / α-6T / TPBI / BPhen / Ag	1.50	1.88	0.38	0.83

Supplementary Table 3. Coulomb binding (E_b) energy and total energy (E_{eh}=IP-EA+E_b) of classical electron-hole (e-h) configurations, with charges localized on different molecules in the standing (S) or in the lying (L) domain. E_b was calculated with the charge response model, as the screened interaction between the electron and the hole sitting on neighboring molecules, while solid-state IP and EA come from embedded *GW* calculations for the "1+1" system. The table reports the mean values over all molecular pairs (within 5 Å intermolecular atom-atom distance), the corresponding standard deviations are in the 30-40 meV range.

	S^+S^-	L^+L^-	S^+L^-	L^+S^-
E_b (eV)	-0.68	-0.64	-0.44	-0.44
E_{eh} (eV)	2.61	2.65	2.43	3.27

Supplementary Table 4. Energies of frontier energy levels (and gap) of a gas-phase α-6T molecule (eV units) obtained at the Kohn-Sham (K-S) level, and from quasiparticle single-iteration (G_0W_0) and self-consistent (on eigenvalues, ev*GW*) *GW* calculations. All calculations are based on K-S orbitals obtained with the gap-tuned ωB97X functional and the cc-pVTZ basis. G_0W_0 results were obtained by correcting only HOMO and LUMO levels. ev*GW* levels were obtained by correcting 8 occupied and 8 unoccupied levels. These results show that a minimal G_0W_0 scheme, used in conjunction with a gap-tuned functional, ensures an accuracy comparable to much more expensive e*vGW* calculations, allowing a reliable description on large systems (up to 264 atoms).

	K-S	G_0W_0	ev*GW*
LUMO+2	0.508	0.734	0.797
LUMO+1	-0.240	-0.014	0.053
LUMO	-0.861	-0.635	-0.580
gap	5.244	5.739	5.805
HOMO	-6.105	-6.374	-6.385
HOMO-1	-6.802	-7.072	-7.103
HOMO-2	-7.722	-7.991	-8.005

Supplementary Table 5. Bethe-Salpeter equation lowest-energy singlet excitation (S_1) of a gas-phase α-6T molecule obtained with different quasiparticle levels, and for different dimensions of the occupied-to-virtual transition space, build with the occupied (unoccupied) levels within the specified cutoff energy from the HOMO (LUMO).

QP levels	Cutoff (eV)	Energy (eV)	f_{osc}
ev*GW*	25	2.871	2.143
ev*GW*	15	2.933	2.130
G_0W_0	25	2.824	2.095
G_0W_0	15	2.883	2.083
G_0W_0	10	2.940	2.083

Supplementary Table 6. Embedded Bethe-Salpeter equation results for the “1+1” system for different dimensions of the occupied-to-virtual transition space, build with the occupied (unoccupied) levels within the specified cutoff energy from the HOMO (LUMO). The table reports the energy and the oscillator strength (between parentheses) of the three lowest-energy excitations (see Supplementary Figure 16).

Cutoff (eV)	S_1	S_2	S_3
10.0	2.374 (1.8772)	2.400 (1.7231)	2.571 (0.0011)
15.0	2.417 (1.8388)	2.448 (1.6978)	2.571 (0.0018)

Supplementary Table 7. Device statistics for the solar cells shown in this work. The devices have been processed by thermal evaporation in vacuum, which is known for its high reproducibility. The reproducibility of the results was tested by fabricating four devices in each case, and from two different batches of α-6T. The data shown in Table 1 of the main text, correspond to the best device (highlighted in bold in the table below) among the four.

Materials	Batch	Device	J_{SC} (mA cm^{-2})	V_{OC} (V)	FF (%)	PCE (%)
α-6T/BPhen	A	**1**	**1.33**	**1.25**	**41.4**	**0.7**
		2	1.32	1.25	41.0	0.7
		3	1.33	1.25	41.3	0.7
		4	1.31	1.25	41.1	0.7
	B	1	1.28	1.25	44.1	0.7
		2	1.26	1.25	43.7	0.7
		3	1.26	1.25	43.8	0.7
		4	1.25	1.25	44.2	0.7
α-6T/TCTA	A	**1**	**3.55**	**1.57**	**39.4**	**2.2**
		2	3.56	1.56	39.7	2.2
		3	3.53	1.56	39.6	2.2
		4	3.49	1.57	39.1	2.1
	B	1	3.45	1.57	38.6	2.1
		2	3.50	1.56	38.1	2.1
		3	3.51	1.57	38.2	2.1
		4	3.48	1.57	38.4	2.1
α-6T/TBPe	A	**1**	**3.00**	**1.41**	**42.5**	**1.8**
		2	3.02	1.41	42.0	1.8
		3	3.03	1.41	42.1	1.8
		4	3.00	1.41	41.9	1.8
	B	1	2.98	1.41	41.9	1.8
		2	3.03	1.41	41.4	1.8
		3	3.05	1.41	41.6	1.8
		4	3.01	1.41	41.8	1.8
α-6T/C545T	A	**1**	**3.82**	**1.46**	**34.7**	**1.9**
		2	3.80	1.48	33.6	1.9
		3	3.79	1.46	33.5	1.8
		4	3.81	1.46	33.3	1.8

	B	1	3.64	1.46	33.1	1.8
		2	3.66	1.46	32.9	1.8
		3	3.70	1.47	32.4	1.8
		4	3.69	1.46	33.4	1.8
α-6T/DBzA	A	1	3.27	1.56	40.6	2.1
		**2**	**3.33**	**1.57**	**39.9**	**2.1**
		3	3.27	1.57	39.8	2.0
		4	3.33	1.56	39.4	2.0
	B	1	3.25	1.56	39.4	2.0
		2	3.32	1.57	39.1	2.0
		3	3.29	1.57	38.9	2.0
		4	3.27	1.57	38.5	2.0
α-6T/TPBI	A	**1**	**3.45**	**1.50**	**33.3**	**1.6**
		2	3.43	1.48	32.9	1.6
		3	3.44	1.50	33.0	1.6
		4	3.41	1.47	33.6	1.6
	B	1	3.33	1.50	32.7	1.6
		2	3.29	1.49	32.9	1.6
		3	3.25	1.48	33.4	1.5
		4	3.30	1.50	33.1	1.6
α-6T/TPBA	A	**1**	**3.61**	**1.61**	**47.0**	**2.8**
		2	3.58	1.60	45.5	2.7
		3	3.62	1.61	45.6	2.8
		4	3.63	1.58	46.3	2.7
	B	1	3.59	1.61	45.5	2.7
		2	3.55	1.60	45.2	2.7
		3	3.52	1.61	46.1	2.7
		4	3.50	1.61	45.9	2.7
α-6T/Rubrene	A	1	3.79	1.61	50.3	2.9
		**2**	**3.80**	**1.61**	**50.2**	**2.9**
		3	3.78	1.61	50.4	2.9
		4	3.75	1.61	50.1	2.8
	B	1	3.62	1.61	49.8	2.8
		2	3.65	1.61	49.5	2.8

		3	3.66	1.61	48.7	2.8
		4	3.63	1.61	48.6	2.8

Supplementary Notes

Supplementary Note 1: Simulation of the α-6T standing/lying interface.

The interface morphology has been built from the experimental crystal structure of the "high-temperature" α-6T polymorph.[16] The standing and lying subsystems were obtained by cutting the crystal along the (0 0 1) and (−1 0 2) planes. The two slabs were assembled face to face as shown in Supplementary Figure 12, as to obtain a sample with an in-plane lattice mismatch lower than 2.2%.

This initial sample has been relaxed with classical simulations based on a force field that has been developed and validated in a previous work.[17] A first molecular dynamics (MD) equilibration at 300 K and 1 atm (NPT ensemble, 3D periodic boundary conditions) has been run for 10 ns to relax atomic positions and cell parameters. Periodic boundary conditions along the z direction were then switched off and atomic coordinates were force-field optimized to remove the effect of thermal motion. Simulations and minimization were performed with the NAMD software.[18]

The quantum sub-system of hybrid quantum/classical (QM/MM) calculations was further optimized at the density functional theory (DFT) level, as to remedy possible inaccuracies of the force field, especially in the description of intramolecular degrees of freedom, that may affect the energetics of electronic excitations. Geometry optimizations were performed at the PBE-D3/def2-SVP level,[19] accounting for the contribution of the MM environment (whose coordinates were kept frozen). QM-MM interactions have been modelled with the same atomic charges and van der Waals parameters adopted in force field simulations. The QM/MM optimization was performed with the ORCA code.[20]

Supplementary Note 2: Electronic structure calculations details.

QM/MM calculations were performed for two different sizes of the QM subsystem, labelled "1+1" (1 standing and 1 lying molecules, 88 QM atoms) and "3+3" (3 standing and 3 lying molecules, 264 QM atoms).

QM/MM *GW*[21] and Bethe-Salpeter equation (BSE)[22] many-body electronic structure calculations were performed with the FIESTA package. *GW* and BSE calculations employed Kohn-Sham orbitals obtained with the specifically gap-tuned density functional ωB97X (gas phase calculations gave an optimal ω=0.141 bohr^{-1}, see Supplementary Figure 13 for details) and the cc-pVTZ basis set. All DFT calculations have been performed with the ORCA code.[20] In many-body calculations, the universal Weigend Coulomb fitting set of functions[23] has been adopted as auxiliary basis in the resolution of the identity (RI-V) scheme.[24] Quasiparticle energy levels were obtained with a single-iteration G_0W_0 scheme with correction on HOMO and LUMO levels only. BSE calculations were performed beyond the Tamm-Dancoff approximation, considering an occupied-to-virtual transition space including the occupied (unoccupied) levels within 10 eV from the HOMO (LUMO). We have explicitly checked that these computational settings ensure an optimal balance between computational accuracy and cost (see Supplementary Tables 4, 5, 6). Electron-hole density plots correspond to the hole-averaged electron density and electron-averaged hole density computed from the two-body BSE eigenstates.

In *GW*/BSE calculations, the MM polarizable crystalline environment has been described at the atomistic level with the charge response model[25] as implemented in the MESCal code[26] Details on embedded *GW* and BSE calculations can be found in previous publications.[21,22] Electrostatic embedding in the ground-state DFT calculation (providing the starting point for the subsequent *GW* treatment) employed a slab geometry, including periodic replica in two dimensions of the MM environment within a cutoff distance of 80 nm (~19 million MM atoms). This ensures the convergence within 50 meV of the electrostatic potential within the QM region. We have explicitly checked that the relative in-plane orientation between the lattices of standing and lying molecules has a minimal effect on the charge carriers energy levels (see Supplementary Figure 17). The reaction field matrix describing the dielectric screening of the electronic excitations within the QM region by the MM environment[21,27] has been evaluated accounting for all MM molecules within a 4 nm distance from the center of the QM region. This cutoff typically ensures the convergence of optical excitations (quasiparticle levels) within 10 meV (100 meV) with respect to an infinite bulk.

Supplementary Note 3: Relative energies of intra and inter-domain CT states.

In order to pinpoint the difference between intra and inter-domain CT states, Supplementary Table 3 reports the energies of localized e-h pairs with charges located either in the same domain or on the two sides of the boundary. These calculations show that intra-layer CT states occur at an energy ~0.2 eV higher than inter-layer ones, as a result of the interplay between the electrostatic landscape and the Coulomb binding energy. The binding energy of intra-layer (S^+S^- and L^+L^-) e-h pairs is ~0.2 eV larger in magnitude than for inter-layer (S^+L^- and L^+S^-) ones, because of the smaller intermolecular distance. This energy difference between intra- and inter-layer e-h configurations is, however, smaller in magnitude than the 0.4 eV offset determined by the electrostatic landscape, so that the lowest-energy intermolecular e-h configurations correspond to inter-layer S^+L^- states. These calculations show that CT excitons in a single domain would be significantly higher in energy, preventing a possible hybridization with low-energy Frenkel molecular excitons, and more Coulombically bound, hampering the charge separation process.

Supplementary References

1. Opitz, A. *et al.* Charge separation at molecular donor-acceptor interfaces: Correlation between morphology and solar cell performance. *IEEE J. Sel. Top. Quantum Electron.* **16**, 1707–1717 (2010).

2. Kahn, A., Koch, N. & Gao, W. Electronic structure and electrical properties of interfaces between metals and π-conjugated molecular films. *J. Polym. Sci. Part B Polym. Phys.* **41**, 2529–2548 (2003).

3. Hiramoto, M. *et al.* Bandgap science for organic solar cells. *Electron.* **3**, 351–380 (2014).

4. Kim, G. W. *et al.* Diphenanthroline electron transport materials for the efficient charge generation unit in tandem organic light-emitting diodes. *Chem. Mater.* **29**, 8299–8312 (2017).

5. Xu, T. *et al.* Highly simplified reddish orange phosphorescent organic light- emitting diodes incorporating a novel carrier- and exciton-confining spiro-exciplex-forming host

for reduced efficiency rolloff. *ACS Appl. Mater. Interfaces* **9**, 2701–2710 (2017).

6. Liu, L., Li, S., Zhou, Y. M., Liu, L. Y. & Cao, X. A. High-current stressing of organic light-emitting diodes with different electron-transport materials. *Microelectron. Reliab.* **71**, 106–110 (2017).

7. Kim, N. H. *et al.* Color optimization of single emissive white OLEDs via energy transfer between RGB fluorescent dopants. *J. Lumin.* **143**, 723–728 (2013).

8. Okumoto, K., Kanno, H., Hamaa, Y., Takahashi, H. & Shibata, K. Green fluorescent organic light-emitting device with external quantum efficiency of nearly 10%. *Appl. Phys. Lett.* **89**, 063504 (2006).

9. Galbadrakh, R., Bang, H., Baek, H. & Lee, C. Three white organic light-emitting diodes with blue-green fluorescent and red phosphorescent dyes. *J. Inf. Disp.* **9**, 23–27 (2008).

10. Cui, R. *et al.* High performance blue and white fluorescent organic electroluminescent devices with conventional electron transport material as blue emitter. *Dye. Pigment.* 108354 (2020) doi:10.1016/j.dyepig.2020.108354.

11. Xu, Z., Tang, B. Z., Wang, Y. & Ma, D. Recent advances in high performance blue organic light-emitting diodes based on fluorescence emitters. *J. Mater. Chem. C* **8**, 2614–2642 (2020).

12. Song, W. & Yook, K. S. Hyperfluorescence-based full fluorescent white organic light-emitting diodes. *J. Ind. Eng. Chem.* **61**, 445–448 (2018).

13. Yu, Y. *et al.* Strategy for achieving efficient electroluminescence with reduced efficiency roll-off: Enhancement of hot excitons spin mixing and restriction of internal conversion by twisted structure regulation using an anthracene derivative. *J. Mater. Chem. C* **7**, 5604–5614 (2019).

14. Vandewal, K., Benduhn, J. & Nikolis, V. C. How to determine optical gaps and voltage losses in organic photovoltaic materials. *Sustainable Energy and Fuels* vol. 2 538–544 (2018).

15. Smilgies, D.-M. Scherrer grain-size analysis adapted to grazing-incidence scattering with area detectors. *J. Appl. Crystallogr.* **42**, 1030–1034 (2009).

16. Siegrist, T. *et al.* The crystal structure of the high-temperature polymorph of α-hexathienyl (α-6T/HT). *J. Mater. Res.* **10**, 2170–2173 (1995).

17. Pizzirusso, A., Savini, M., Muccioli, L. & Zannoni, C. An atomistic simulation of the liquid-crystalline phases of sexithiophene. *J. Mater. Chem.* **21**, 125–133 (2011).

18. Phillips, J. C. *et al.* Scalable molecular dynamics with NAMD. *J. Comput. Chem.* **26**, 1781–1802 (2005).

19. Grimme, S., Ehrlich, S. & Goerigk, L. Effect of the damping function in dispersion corrected density functional theory. *J. Comput. Chem.* **32**, 1456–1465 (2011).

20. Angeli, C., Bories, B., Cavallini, A. & Cimiraglia, R. Third-order multireference perturbation theory: The n-electron valence state perturbation-theory approach. *J. Chem. Phys.* **124**, 054108 (2006).

21. Li, J., D'Avino, G., Duchemin, I., Beljonne, D. & Blase, X. Accurate description of charged excitations in molecular solids from embedded many-body perturbation theory. *Phys. Rev. B* **97**, 1–13 (2018).

22. Duchemin, I., Guido, C. A., Jacquemin, D. & Blase, X. The Bethe-Salpeter formalism with polarisable continuum embedding: Reconciling linear-response and state-specific features. *Chem. Sci.* **9**, 4430–4443 (2018).

23. Weigend, F. Accurate Coulomb-fitting basis sets for H to Rn. *Phys. Chem. Chem. Phys.* **8**, 1057 (2006).

24. Duchemin, I., Li, J. & Blase, X. Hybrid and Constrained Resolution-of-Identity Techniques for Coulomb Integrals. *J. Chem. Theory Comput.* **13**, 1199–1208 (2017).

25. Tsiper, E. V. & Soos, Z. G. Charge redistribution and polarization energy of organic molecular crystals. *Phys. Rev. B - Condens. Matter Mater. Phys.* **64**, 1–12 (2001).

26. D'Avino, G., Muccioli, L., Zannoni, C., Beljonne, D. & Soos, Z. G. Electronic Polarization in Organic Crystals: A Comparative Study of Induced Dipoles and Intramolecular Charge Redistribution Schemes. *J. Chem. Theory Comput.* **10**, 4959–4971 (2014).

27. Li, J., D'Avino, G., Duchemin, I., Beljonne, D. & Blase, X. Combining the Many-Body GW Formalism with Classical Polarizable Models: Insights on the Electronic Structure of Molecular Solids. *J. Phys. Chem. Lett.* **7**, 2814–2820 (2016).
