# Supplementary material for: Orientation dependent molecular electrostatics drives efficient charge generation in homojunction organic solar cells
Source: Nat Commun. 2020 Sep 15;11:4617. doi: 10.1038/s41467-020-18439-z (PMC7494863; doi:10.1038/s41467-020-18439-z)
Supplement: Supplementary file 3 — Reporting Summary [file 41467_2020_18439_MOESM3_ESM.pdf]

Corresponding author(s): Koen Vandewal, James R. Durrant, Gabriele D'Avino, Ji-Seon Kim

Solar Cells Reporting Summary

Nature Research wishes to improve the reproducibility of the work that we publish. This form is intended for publication with all accepted papers reporting the characterization of photovoltaic devices and provides structure for consistency and transparency in reporting. Some list items might not apply to an individual manuscript, but all fields must be completed for clarity.

For further information on Nature Research policies, including our data availability policy, see Authors & Referees.

ü Experimental design

Please check: are the following details reported in the manuscript?

1. Dimensions

Item	Yes	No	Details
Area of the tested solar cells	☒	☐	Details are given in the Methods section.
Method used to determine the device area	☒	☐	Details are given in the Methods section.

2. Current-voltage characterization

Item	Yes	No	Details
Current density-voltage (J-V) plots in both forward and backward direction	☐	☒	Since no hysteresis is reported for small molecule organic solar cells, J-V measurements in both directions are not needed.
Voltage scan conditions *For instance: scan direction, speed, dwell times*	☐	☒	Since no hysteresis is reported for small molecule organic solar cells, scan direction, speed or dwell times are not relevant.
Test environment *For instance: characterization temperature, in air or in glove box*	☒	☐	Details are given in the Methods section.
Protocol for preconditioning of the device before its characterization	☒	☐	Since no hysteresis is reported for small molecule organic solar cells, no precinditioning is required.
Stability of the J-V characteristic *Verified with time evolution of the maximum power point or with the photocurrent at maximum power point; see ref. 7 for details.*	☐	☒	Small molecule organic solar cells are known to be stable.

3. Hysteresis or any other unusual behaviour

Item	Yes	No	Details
Description of the unusual behaviour observed during the characterization	☐	☒	No unusual behavior was observed.
Related experimental data	☐	☒	Not needed, see above.

4. Efficiency

Item	Yes	No	Details
External quantum efficiency (EQE) or incident photons to current efficiency (IPCE)	☒	☐	In the main text and the supplementary information.
A comparison between the integrated response under the standard reference spectrum and the response measure under the simulator	☐	☒	J-V measurements are performed with mismatch corrected illumination spectra. Details are given in the Methods section
For tandem solar cells, the bias illumination and bias voltage used for each subcell	☐	☒	No tandem solar cells are included in this work.

5. Calibration

Item	Yes	No	Details
Light source and reference cell or sensor used for the characterization	☒	☐	Details are given in the Methods section.
Confirmation that the reference cell was calibrated and certified	☒	☐	Details are given in the Methods section.

Calculation of spectral mismatch between the reference cell and the devices under test	[x] Yes [ ] No	Details are given in the Methods section.

6. Mask/aperture

Size of the mask/aperture used during testing	[x] Yes [ ] No	Details are given in the Methods section.
Variation of the measured short-circuit current density with the mask/aperture area	[ ] Yes [x] No	Not relevant for the scope of this work.

7. Performance certification

Identity of the independent certification laboratory that confirmed the photovoltaic performance	[ ] Yes [x] No	A certified efficiency is not relevant for the scope of this work.
A copy of any certificate(s)<br>*Provide in Supplementary Information*	[ ] Yes [x] No	No certificate. Please see above.

8. Statistics

Number of solar cells tested	[x] Yes [ ] No	Each fabricated sample contains 4 different pixels, which showed similar performance. The best pixel was selected and shown in this work.
Statistical analysis of the device performance	[ ] Yes [x] No	Vacuum processed organic solar cells are known to be highly reproducible.

9. Long-term stability analysis

Type of analysis, bias conditions and environmental conditions<br>*For instance: illumination type, temperature, atmosphere humidity, encapsulation method, preconditioning temperature*	[ ] Yes [x] No	All devices were encapsulated. Moreover, small molecule organic solar cells are known to be stable. Long-term stability measurements are not in the scope of this work.
